# Highly integrated bionic prostheses resolve the thermal asymmetry between residual amputated and contralateral limbs

**DOI:** 10.1038/s41598-023-33210-2

**Published:** 2023-04-17

**Authors:** Victoria Ashley Lang, Maria Munoz-Novoa, Max Ortiz-Catalan

**Affiliations:** 1 Center for Bionics and Pain Research, Mölndal, Sweden; 2 Department of Electrical Engineering, Chalmers University of Technology, Gothenburg, Sweden; 3 Department of Physiology, Institute of Neuroscience and Physiology, Sahlgrenska Academy, University of Gothenburg, Gothenburg, Sweden; 4 Center for Advanced Reconstruction of Extremity, Sahlgrenska University Hospital, Mölndal, Sweden; 5 Bionics Institute, Melbourne, Australia

**Keywords:** Biomedical engineering, Neuroscience

## Abstract

Residual limbs after amputation present colder temperatures than unaffected contralateral limbs. This temperature asymmetry has been attributed to autonomic and cognitive factors, such as changes in body representation. An ideal limb replacement should restore the body representation and resolve the temperature asymmetry, but conventional prostheses, commonly characterized as disembodied, fail to do so. Neuromusculoskeletal prostheses are a new concept of artificial limbs that directly interface with the user’s nerves, muscles, and skeleton, and are operated in daily life by bidirectionally transferring control and somatosensory information. Here, we show that the temperature asymmetry commonly found in people with amputations is resolved when using a neuromusculoskeletal prosthesis but reappears when it is removed. A potential explanation for this phenomenon might be the increased embodiment reported by users of neuromusculoskeletal prostheses, which in turn would suggest unconscious perceptual mechanisms mediating the temperature asymmetry commonly found between intact and residual limbs after amputation.

## Introduction

Symmetry in body temperature is a notion rarely doubted and a general marker of good health. Thermal recordings of healthy humans have indicated that a lateral difference greater than 1 °C is anomalous, especially over the torso and upper regions of the limbs^1,2^. Temperature measurements of the digits may occasionally reveal asymmetries greater than 1 °C, but this variability can be explained by the role that the extremities play in thermal regulation^2^. Detecting abnormalities in thermal profiles have been useful in prescreening for breast cancers and evaluating risks for the development of vascular diseases^3–5^.

In unilateral amputations, the distal residual limb has a significantly colder skin temperature than the intact contralateral limb^6^. Theories surrounding this temperature difference include distortions in autonomic regulation^6,7^ and disembodiment of the missing limb^8,9^, but the underlying mechanisms continue to be debated. Nevertheless, the degree of normalization of residual limb temperature could be used as a biomarker and objective measurement of the integration of artificial limbs.

A neuromusculoskeletal interface provides direct skeletal attachment to the residual bone, while also allowing for bidirectional connection to nerves and muscles to extract control signals and to deliver intuitive sensory feedback via neurostimulation^10,11^. Direct skeletal attachment via osseointegration avoids problems associated with conventional suspension sockets, such as discomfort and abrasions^12,13^, which reduces prosthetic use. The neuromusculoskeletal interface allows for the long-term and uninterrupted use of sensate prosthetic limbs^10,11^, thereby resulting in subjective reports of embodiment of the artificial replacement^14^.

**Figure 1:**
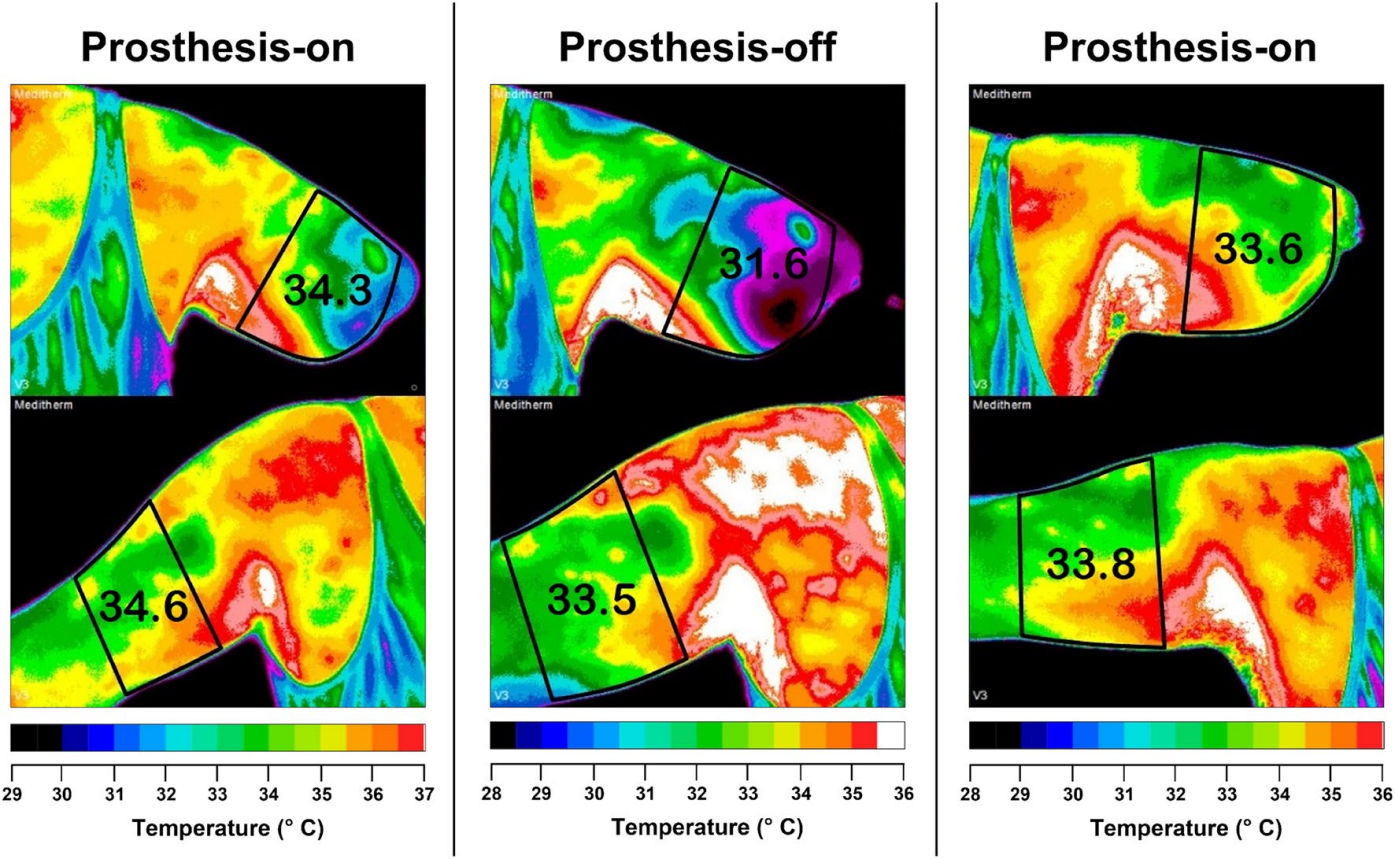
Neuromusculoskeletal prosthesis users had thermal images taken three times on two consecutive days: (1) Prosthesis-on, (2) Prosthesis-off for 15.72 h (SD = 1.58), and (3) Prosthesis-on again for 7.22 h (SD = 1.82). The lateral temperature differences across the three columns are 0.3 °C, 1.9 °C, and 0.2 °C, in this example.

We recorded the skin temperature of the residual and contralateral limbs using an infrared camera tuned for physiological ranges (0.01 °C measurement sensitivity). Infrared thermography is a non-invasive technique that produces greater accuracy and reproducibility compared to other methods for collecting thermal measurements^6,15^. This technology has played an important role in identifying patterns in disease states, assisting surgical procedures, and indicating sympathetic and local vasoactive tone^7,16–22^. For instance, pain intensity and quality have been found to correspond to changes in surface blood flow^23^. We found that wearing neuromusculoskeletal, but not conventional prostheses, reduces the skin temperature asymmetry between the amputated and contralateral limb, and that said asymmetry increases when the neuromusculoskeletal prosthesis is removed (Fig. 1). Our finding suggests skin temperature asymmetry as a potential biomarker for the integration of artificial limbs.

## Results

Four people with unilateral transhumeral amputations implanted with neuromusculoskeletal prostheses participated in this study. We examined the skin temperature changes from wearing a prosthesis (prosthesis-on) to removing the prosthesis (prosthesis-off), and then wearing it again (prosthesis-on). Participants wore their neuromusculoskeletal prostheses upon arrival until the first image was taken to fulfill the prosthesis-on condition, then did not wear it for 15.72 h (SD = 1.58) in the prosthesis-off condition, to finalize with at least six hours wearing the prosthesis again for 7.22 h (SD = 1.82). The skin temperature measurement of the residual limb included the area with perimeter requiring the midline of the last distal intact joint and a line proximal to any scar tissue at the amputation site. This corresponding area of the contralateral limb was also measured for comparison (example in Fig. 1).

**Figure 2:**
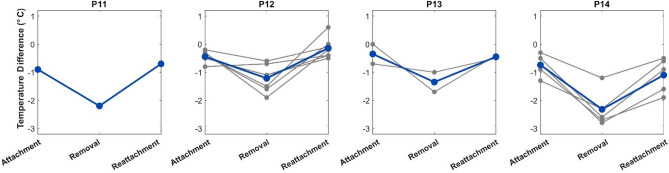
Changes in temperature asymmetry: Neuromusculoskeletal prosthesis-users show a smaller temperature difference between the residual and contralateral limbs when wearing their prostheses. For each participant, the grey lines indicate measurements of all image sets; the blue line indicates the average of all image sets.

We computed the mean temperature of each limb for every participant, and then the mean across all participants, thereby eliminating biasing to participants that participated more than once. The average skin temperature asymmetry under the first prosthesis-on condition was 0.61 °C (SD = 0.22). After the neuromusculoskeletal prosthesis was removed (prosthesis-off condition), the skin temperature of the residual limb decreased to 1.77 °C (SD = 0.49) in comparison to the contralateral limb. This difference was then reduced back to 0.60 °C (SD = 0.35) after the participants wore their neuromusculoskeletal prosthesis again (Fig. 2). Paired-sampled t-tests determine that the two prosthesis-on datasets do not statistically differ (*P* > 0.95, N = 4) and the prosthesis-on and -off datasets statistically differ (*P* < 0.01, N = 4).

**Figure 3:**
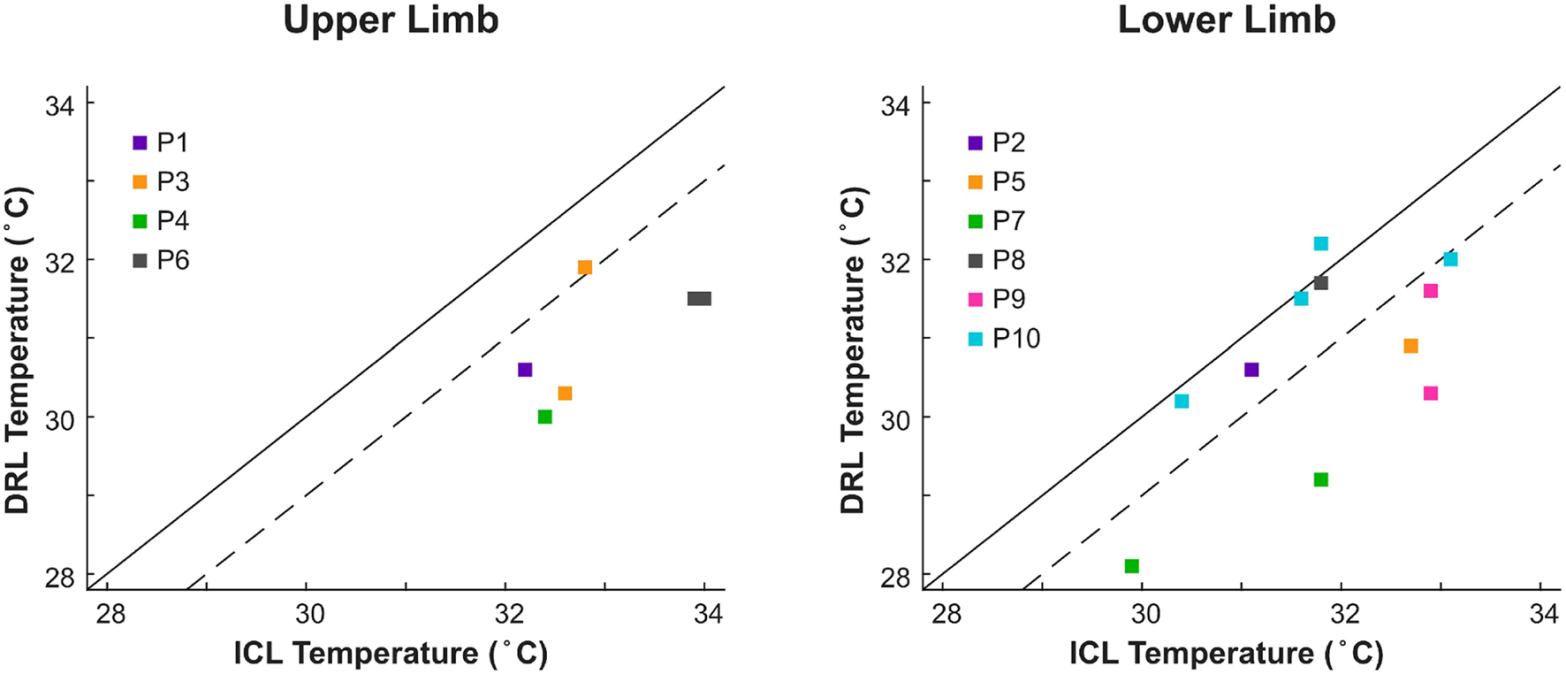
Skin temperature asymmetry: Intact contralateral limb (ICL) versus distal residual limb (DRL) temperature on conventional prostheses-users. Solid line corresponds to thermal symmetry between the limbs. Dashed line corresponds to DRL 1 ° C cooler than ICL.

We verified our methods by measuring the skin temperature asymmetry in ten participants with unilateral amputation who utilized conventional prostheses. Five participants were measured once, four participated twice, and one participated four times, giving a total of 17 image sets (Fig. 3). Like in the neuromusculoskeletal prostheses group, we computed the mean temperatures for each participant, and then calculated the mean across all participants in the conventional prostheses group. All image sets indicated a colder residual limb, except for in one set. The inconsistent set belonged to participant P10 who completed four sessions, in which the other three images showed a colder residual limb.

The mean skin temperature of the residual limb was 30.75 °C (SD = 0.85), while the mean temperature of the contralateral limb was 32.23 °C (SD = 0.87). A paired-samples t-test determined that the mean temperature of the residual limb was significantly colder than the mean temperature of the contralateral (*P* < 0.01, N = 10) with an average difference of 1.49 °C (SD = 0.84).

## Discussion

A healthy individual is said to have a bilateral temperature difference of no larger than 1 °C between the upper extremities and core regions of the body^1,2^. In general, people with amputations have a skin temperature asymmetry exceeding 1 °C, indicative of a potential pathophysiology. The mechanism of this temperature asymmetry after limb amputation is unknown^24,25^.

We verified our method for measuring skin temperature asymmetry by confirming previous findings of larger than 1 °C difference in people with unilateral amputations wearing conventional socket prostheses (upper and lower limbs). We found the residual limb was on average 1.49 °C (SD = 0.84) colder than the contralateral, which is in agreement with previous work reporting an average difference of 1.1 °C^6^. Users of conventional socket prostheses were subject to a single image measurement because it has been previously reported that intra-day temperature variations are symmetric across the body^1,2^.

Using the same thermal imagining technology, we observed a shift towards bilateral temperature symmetry in users of neuromusculoskeletal prostheses. Temperature asymmetry in these participants was less than half of those without the neuromusculoskeletal interface investigated in here and in work by others^6^. More importantly, removing the neuromusculoskeletal prosthesis exacerbated the asymmetry to values similar to the participants using conventional prostheses, thus indicating causality. Interestingly, the changes in temperature after donning and doffing of the neuromusculoskeletal prostheses were not immediate. In preliminary experiments, we attempted to capture such changes by taking thermal pictures every five minutes during 30 min after removing or wearing the prosthesis but found no measurable change. We therefore decided to utilize spans of six hours.

The neuromusculoskeletal interface allows for more reliable, responsive, and precise prosthetic control than conventional non-invasive technologies^26^. People with neuromusculoskeletal prostheses have shown improved grasping function, particularly under uncertainty, thanks to the provision of somatosensation elicited via direct neural stimulation^27^, which lacks in conventional prostheses. The difference in control and somatosensation between neuromusculoskeletal and conventional prostheses results in a higher degree of agency and ownership^14^, both conclusive to prosthetic embodiment^28,29^. In addition, the superior comfort provided by direct skeletal attachment via osseointegration, as opposed to socket suspension, allows for patients to wear their prosthesis all day and every day^10,11,30^. Overall, living with such an integrated human–machine interface has shown to have positive social and psychological effects in the users, who see these prostheses as part of their bodies^14^. Embodiment of the prosthesis could be a potential reason for the reduction in temperature asymmetry, as subconscious changes in the body representation have been thought to cause an optimization of autonomic efforts to supply blood to parts of the body no longer present. Restoration of the body representation by an embodied prosthetic limb might reverse said optimization, restoring in turn the temperature in the residual limb. Based on this hypothesis, we propose skin temperature asymmetry as an objective biomarker for artificial limb integration.

Despite the improvements reported by neuromusculoskeletal prostheses, this technology is still far from equating a biological limb. At present, they provide limited control and sensory feedback^31,32^, which may explain why there is still certain level of temperature asymmetry even in users of these more integrated prostheses. Further improvements to this, or any other technology for artificial limb replacement, could be potentially evaluated by the symmetry of skin temperature.

A limitation of this study is that recordings of skin temperature were not taken on the participants with neuromusculoskeletal prostheses prior being implanted. This prevented a direct comparison within each participant before and after implantation. We therefore adopted a protocol in which the prosthesis was removed and worn again to test causality. The absence of the prosthesis showed a reduction in skin temperature of the residual limb, which indicates that the presence of the implant itself was not enough to maintain temperature symmetry. We must yet study what components of using a neuromusculoskeletal prosthesis contribute primarily to this phenomenon, as prosthetic control has a direct impact on the sense of agency, and sensory feedback on the sense of ownership.

In our preliminary work, we found that skin temperature changes in users of neuromusculoskeletal prostheses could not be observed within 30 min, and thus decided to employ longer periods of time. We allowed for at least six hours in each condition considering that the longer the time in each condition, the more likely would be for the steady state to have settled. Therefore, one could argue that periods of days instead of hours would allow for stronger effects. Nevertheless, we observed that at least 6 h of wearing the prosthesis again was enough to reduce skin temperature asymmetry to values close to those prior removal. Another potential limitation of this study is that measurements were taken at different times of the days in which skin temperature might have varied (morning and evenings). Whereas this is true for the absolute value of skin temperature, our interest was on the difference between the residual and contralateral limbs, which is a relative value observed to be salient regardless of the time of the day^1^.

## Methods

### Participants

All participants signed an informed consent form approved by Swedish Ethical Review Authority (Dnr 2019–05,448). Data privacy and management complied with the EU General Data Protection Regulation 2016/679 (GDPR). All research was performed in accordance with the Declaration of Helsinki and the relevant guidelines and regulations. This study was approved by the Ethics Review Authority (Etikprövningsmyndigheten) in Sweden.

Fourteen unilateral amputees using a prosthesis in daily life were recruited for this study, of which 12 were male and 2 were female. Their ages ranged from 18 to 79 with an average age of 48.9 (SD = 14.9). The four participants with the neuromusculoskeletal interface used a commercially available elbow (myoelectric locking) and a one-degree of freedom myoelectric hand. Three of the four patients had sensory feedback enable via direct nerve stimulation. Nine participants used passive prostheses attached with a socket, and one participant did not use and had never used any prosthesis. Additional details are given in Table 1.

**Table 1:** Participant data.

Participant	Type of prosthesis	Sex	Age in 2020	Amputated limb	Dominant side before amputation
P1	Socket	M	18	Right arm	Not reported
P2	Socket	M	36	Right leg	Not reported
P3	Socket	M	79	Right arm	Left
P4	Socket	M	46	Right arm	Not reported
P5	Socket	M	75	Right leg	Right
P6	None	M	45	Left arm	Right
P7	Socket	M	59	Right leg	Right
P8	Socket	M	56	Right leg	Right
P9	Socket	F	40	Right leg	Right
P10	Socket	F	39	Right leg	Right
P11	Neuromusculoskeletal	M	47	Left arm	Right
P12	Neuromusculoskeletal	M	52	Left arm	Right
P13	Neuromusculoskeletal	M	45	Right arm	Right
P14	Neuromusculoskeletal	M	48	Right arm	Right

### Temperature asymmetry measurement

Since wearing a socket could affect the residual limb temperature, we chose an acclimatization period after removing the socket of 15 min prior to thermal imaging^6,33^. This acclimatization period reduced the possibility of detecting an unusually warm residual limb due to liner wear, compression, or abrasions from socket. Images for upper-limb amputees were taken while they were facing forward, arms held laterally away from the torso, and palms facing the camera. Lower-limb amputees were asked to stand face forward with assistance.

*Measurement of conventional prostheses users*: Socket prosthesis-users and the single amputee without any prosthesis participated in 1–4 imaging sessions consisting of 1 image to document the temperatures of their residual and contralateral limbs.

*Measurement of neuromusculoskeletal prostheses users*: Neuromusculoskeletal prosthesis-users participated in 1–7 imaging sessions over two days consisting of 3 images: (1) At the end of day 1, (2) At the start of day 2, and (3) At the end of day 2. Participants were required to freely use their prosthesis during the day and remove their prosthesis overnight without reattachment before the 2nd image.

### Equipment and analysis

Thermal images were acquired using the Meditherm Iris 380 (Meditherm, Cheyenne, USA) camera with measurement sensitivity of 0.01 °C. The camera was controlled with Meditherm WinTES3 camera software on a personal computer. Using the same software, the thermal images were analyzed by “drawing” a polygon outline on the anterior surface of the DRL. The proximal limit of the polygon drawn on the DRL was the midline of the last distal intact joint and the distal limit was a line proximal to any scar tissue at the amputation site. A similar polygon was drawn on the corresponding area of the ICL. All drawings were completed by a single researcher to avoid differences in drawing technique.

## Data Availability

All data presented in this study is available upon reasonable request to the corresponding/senior author.

## References

[1] Silberstein EB, Bahr GK, Kattan J (1975). Thermographically measured normal skin temperature asymmetry in the human male. Cancer.

[2] Redisch W, Sheckman E, Steele JM (1952). Skin temperature response of normal human subjects to various conditions. Circulation.

[3] Dodd GD, Zermeno A, Marsh L, Boyd D, Wallace JD (1969). New developments in breast thermography. High spatial resolution. Cancer.

[4] Philip J (2009). Infrared thermal imaging for detection of peripheral vascular disorders. J. Med. Phys..

[5] Huang C-L (2011). The application of infrared thermography in evaluation of patients at high risk for lower extremity peripheral arterial disease. J. Vasc. Surg..

[6] Harden RN, Gagnon CM, Gallizzi M, Khan AS, Newman D (2008). Residual limbs of amputees are significantly cooler than contralateral intact limbs. Pain Pract..

[7] Uematsu S, Edwin DH, Jankel WR, Kozikowski J, Trattner M (1988). Quantification of thermal asymmetry. J. Neurosurg..

[8] Moseley GL (2008). Psychologically induced cooling of a specific body part caused by the illusory ownership of an artificial counterpart. Proc. Natl. Acad. Sci..

[9] Marasco PD, Kim K, Colgate JE, Peshkin MA, Kuiken TA (2011). Robotic touch shifts perception of embodiment to a prosthesis in targeted reinnervation amputees. Brain.

[10] Ortiz-Catalan M, Hakansson B, Branemark R (2014). An osseointegrated human-machine gateway for long-term sensory feedback and motor control of artificial limbs. Sci. Transl. Med..

[11] Ortiz-Catalan M, Mastinu E, Sassu P, Aszmann O, Brånemark R (2020). Self-contained neuromusculoskeletal arm prostheses. N. Engl. J. Med..

[12] Klute GK, Huff E, Ledoux WR (2014). Does activity affect residual limb skin temperatures?. Clin. Orthop. Relat. Res..

[13] Segal AD, Klute GK (2016). Residual limb skin temperature and thermal comfort in people with amputation during activity in a cold environment. J. Rehabil. Res. Dev..

[14] Middleton A, Ortiz-Catalan M (2020). Neuromusculoskeletal arm prostheses: Personal and social implications of living with an intimately integrated bionic arm. Front. Neurorobot..

[15] Gatt A (2015). Thermographic patterns of the upper and lower limbs: Baseline data. Int. J. Vasc. Med..

[16] Pirtini CM, Herman C (2011). Quantification of the thermal signature of a melanoma lesion. Int. J. Therm. Sci..

[17] Romanò CL, Romanò D, Dell’Oro F, Logoluso N, Drago L (2011). Healing of surgical site after total hip and knee replacements show similar telethermographic patterns. J. Orthop. Traumatol..

[18] Di Carlo A (1995). Thermography and the possibilities for its applications in clinical and experimental dermatology. Clin. Dermatol..

[19] Glehr M (2011). Thermal imaging as a noninvasive diagnostic tool for anterior knee pain following implantation of artificial knee joints. Int. J. Thermodyn..

[20] Ohsawa S, Inamori Y, Fukuda K, Hirotuji M (2001). Lower limb amputation for diabetic foot. Arch. Orthop. Trauma Surg..

[21] McCollum PT, Spence VA, Walker WF, Murdoch G (1985). A rationale for skew flaps in below-knee amputation surgery. Prosthet. Orthot. Int..

[22] Piersol JSB, A G (2000). Random data analysis and measurement procedures. Meas. Sci. Technol..

[23] Sherman RA, Bruno GM (1987). Concurrent variation of burning phantom limb and stump pain with near surface blood flow in the stump. Orthopedics.

[24] Kristen H, Lukeschitsch G, Plattner F, Sigmund R, Resch P (1984). Thermography as a means for quantitative assessment of stump and phantom pains. Prosthet. Orthot. Int..

[25] Katz, J. The Role of the Sympathetic Nervous System in Phantom Pain. in *Phantom Pain* 63–88 (Springer US, 1997). 10.1007/978-1-4757-6169-6_4.

[26] Mastinu E (2019). Grip control and motor coordination with implanted and surface electrodes while grasping with an osseointegrated prosthetic hand. J. Neuroeng. Rehabil..

[27] Mastinu E (2020). Neural feedback strategies to improve grasping coordination in neuromusculoskeletal prostheses. Sci. Rep..

[28] Zbinden J, Lendaro E, Ortiz-Catalan M (2022). Prosthetic embodiment: Systematic review on definitions, measures, and experimental paradigms. J. Neuroeng. Rehabil..

[29] Zbinden J, Lendaro E, Ortiz-Catalan M (2022). A multi-dimensional framework for prosthetic embodiment: A perspective for translational research. J. Neuroeng. Rehabil..

[30] Jönsson S, Caine-Winterberger K, Brånemark R (2011). Osseointegration amputation prostheses on the upper limbs: methods, prosthetics and rehabilitation. Prosthet. Orthot. Int..

[31] Ortiz-Catalan M, Wessberg J, Mastinu E, Naber A, Branemark R (2019). Patterned stimulation of peripheral nerves produces natural sensations with regards to location but not quality. IEEE Trans. Med. Robot. Bionics.

[32] Ortiz-Catalan M, Mastinu E, Greenspon CM, Bensmaia SJ (2020). Chronic use of a sensitized bionic hand does not remap the sense of touch. Cell Rep..

[33] Peery JT, Ledoux WR, Klute GK (2005). Residual-limb skin temperature in transtibial sockets. J. Rehabil. Res. Dev..

